# 放疗联合免疫治疗在不可切除III期非小细胞肺癌中的临床研究进展及未来展望

**DOI:** 10.3779/j.issn.1009-3419.2025.102.42

**Published:** 2025-11-20

**Authors:** TAN Zuoyuan, ZHONG Yuling, WANG Jingyi, WU Lin

**Affiliations:** ^1^421001 衡阳，南华大学衡阳医学院湖南省肿瘤医院研究生协作培养基地（谭左源，钟雨玲）; ^1^Graduate Collaborative Training Base of Hunan Cancer Hospital, University of South China, Hengyang Medical School, Hengyang 421001, China; ^2^410013 长沙，湖南省肿瘤医院/中南大学湘雅医学院附属肿瘤医院胸内二科 （谭左源，钟雨玲，王婧怡，邬麟）; ^2^The Second Department of Thoracic Oncology, Hunan Cancer Hospital/The Affiliated Cancer Hospital of Xiangya School of Medicine, Central South University, Changsha 410013, China

**Keywords:** 肺肿瘤, 放疗, 免疫治疗, 免疫检查点抑制剂, 生物标志物, Lung neoplasms, Radiotherapy, Immunotherapy, Immune checkpoint inhibitors, Biomarkers

## Abstract

III期非小细胞肺癌（non-small cell lung cancer, NSCLC）约占初诊NSCLC的30%，且绝大多数患者无法手术切除，其不可手术特性使根治性放化疗成为核心治疗手段。近年来，随着免疫检查点抑制剂（immune checkpoint inhibitors, ICIs）成为肺癌领域的研究热点，越来越多的研究表明放疗联合免疫治疗（combination of radiotherapy and immunotherapy, iRT）能通过协同机制显著提升抗肿瘤疗效，PACIFIC研究的突破性进展更确立了同步放化疗后免疫巩固治疗的标准地位。然而，最佳放疗策略、免疫介入时机、不良事件管理和生物标志物探索等问题仍存争议。本综述旨在系统阐述iRT的协同作用机制，梳理不同iRT治疗模式下（同步、巩固、诱导）的临床试验进展，并对当前临床实践中的关键问题与未来研究方向进行深入探讨。

## Abstract (en)

Stage III non-small cell lung cancer (NSCLC) accounts for approximately 30% of newly diagnosed NSCLC cases, and the vast majority of these patients present with unresectable disease. Its unresectable nature makes definitive chemoradiotherapy the cornerstone of treatment. In recent years, with immune checkpoint inhibitors (ICIs) becoming a major research focus in lung cancer, increasing evidence demonstrates that the combination of radiotherapy and immunotherapy (iRT) can significantly enhance antitumor efficacy through synergistic mechanisms. The groundbreaking results of the landmark PACIFIC trial further established consolidation immunotherapy following concurrent chemoradiotherapy as the standard of care. However, controversies persist regarding optimal radiotherapy strategies, timing of immune intervention, management of adverse reactions, and exploration of biomarkers. This review aims to systematically elucidate the synergistic mechanisms of iRT, summarize clinical trial advances under different iRT treatment modalities (concurrent, consolidation and induction), and provide an in-depth analysis of key issues in current clinical practice along with future research directions.

既往数据^[1]^表明，肺癌是全球发病率和死亡率最高的恶性肿瘤，非小细胞肺癌（non-small cell lung cancer, NSCLC）约占85%。其中，III期NSCLC虽未有远处转移而存在潜在根治可能，但又因为局部侵犯明显、区域淋巴结转移等因素导致无法进行根治性手术，使治疗非常具有挑战性，也成为临床研究的热点和难点。以PACIFIC系列试验为代表的诸多临床试验均证实，放疗联合免疫治疗（combination of radiotherapy and immunotherapy, iRT）具有不同于经典方案的广阔前景和深入挖掘空间。多项研究^[2,3]^表明，iRT能通过促进远隔效应、增强肿瘤免疫原性及重塑免疫抑制性肿瘤微环境（tumor mircroenvironment, TME）等多重协同机制，增强抗肿瘤疗效。但最佳放疗剂量及分割模式、免疫检查点抑制剂（immune checkpoint inhibitors, ICIs）的介入时机、不良事件管理和生物标志物探索等关键问题仍需进一步探索。本综述旨在通过阐明iRT的协同作用机制、重点梳理不同iRT模式下（同步、巩固、诱导）的临床研究进展，深入探讨iRT面临的关键问题并进行未来展望，为iRT在不可切除III期NSCLC中的临床应用提供参考。

## 1 iRT的协同作用机制

### 1.1 促进远隔效应

放疗不仅能局部杀伤照射野内的肿瘤细胞，还能诱导未经照射的远端病灶消退，这一现象被称为远隔效应^[4]^。单纯放疗的远隔效应较为罕见^[5]^，而iRT能显著提升其发生率^[2,4]^。其核心机制在于放疗激活局部效应T细胞免疫应答，ICIs解除全身性免疫抑制状态，促进活化的效应T细胞迁移与浸润至远端病灶，最终实现原发灶与转移灶的协同控制^[6]^。

### 1.2 增强肿瘤免疫原性

肿瘤细胞通过下调抗原呈递机制形成低免疫原性的“冷肿瘤”TME，进而逃避免疫监视^[7]^。放疗可以通过诱导免疫原性细胞死亡（immunogenic cell death, ICD），促进损伤相关的分子模式（daamge-associated molecular pattern, DAMP）和肿瘤相关抗原（tumor associated antigen, TAA）的释放，有效促进抗原加工与交叉呈递，进而增强肿瘤免疫原性^[7,8]^。同时，放疗可以上调主要组织相容性复合体I类（major histocompatibility complex I, MHC-I）等关键表型，并促进黏附分子与趋化因子等表达，进而增强T细胞浸润和招募肿瘤浸润淋巴细胞，提升免疫系统识别和清除肿瘤细胞的能力，有助于将“冷肿瘤”转变为“热肿瘤”^[9-11]^。

### 1.3 免疫抑制性TME

放疗对TME具有双向调节作用。另一方面，放疗会上调肿瘤细胞程序性细胞死亡配体1（programmed cell death ligand 1, PD-L1）表达，促进免疫抑制性细胞的瘤内浸润，并诱导浸润T细胞耗竭，从而导致适应性免疫抵抗^[12,13]^。此时，ICIs的应用至关重要。ICIs可以有效阻断此通路，同时促进肿瘤血管正常化、改善TME的低氧状态，从而协同逆转放疗诱导的免疫抑制性TME，增强整体的协同抗肿瘤反应^[14]^。

## 2 iRT在不可切除III期NSCLC中的研究进展

目前，多项大型临床研究聚焦于不可切除III期NSCLC患者，探索在含铂化疗背景基础上，以iRT为核心的治疗模式的疗效与安全性。本文将根据不同的iRT模式（同步、巩固、诱导等），分别阐述当前研究进展。部分研究的关键试验数据见表1^[15-29]^，正在开展的相关研究见表2^[30-35]^。

**表 1:** 不可切III期NSCLC放疗联合免疫已完成临床试验

Trial	Phase	Interventions	mPFS (mon)	mOS (mon)
Consolidation immunotherapy after chemoradiotherapy				
PACIFIC^[15,16]^	III	cCRT → Durvalumab vs cCRT → placebo	16.9 vs 5.6 HR=0.52, P<0.0001	47.5 vs 29.1 HR=0.68, *P*=0.00251
LUN 14-179^[17]^	II	cCRT → Pembrolizumab	18.7	35.8
GEMSTONE-301^[18]^	III	cCRT/sCRT → Sugemalimab vs cCRT/sCRT → placebo	9.0 vs 5.8 HR=0.64, *P*=0.0026	-
PACIFIC-6^[19]^	II	sCRT → Durvalumab	13.1	39.0
PACIFIC-R^[20]^	III	cCRT/sCRT → Durvalumab	24.3 (mrwPFS)	59.0
COAST^[21,22]^	II	cCRT → Durvalumab+Oleclumab vs cCRT → Durvalumab+Monalizumab vs cCRT → Durvalumab	21.1 vs 19.8 vs 7.3	NR vs NR vs 40.9
Concurrent immunotherapy combined with chemoradiotherapy				
ETOP NICOLAS^[23]^	II	cCRT+Nivolumab → Nivolumab	12.7	38.8
DETERRED^[24]^	II	cCRT → Atezolizumab vs cCRT+Atezolizumab → Atezolizumab	18.9 vs 15.1 HR=1.30, *P*=0.54	26.5 vs NR HR=0.71, *P*=0.51
KEYNOTE-799^[25]^	II	cCRT+Pembrolizumab → Pembrolizumab (squamous±nonsquamous)	29 vs 45.3	35.6 vs 56.7
Induction immunotherapy before chemoradiotherapy				
AFT-16^[26]^	II	Atezolizumab → cCRT → Atezolizumab	30.0	NR
APOLO^[27]^	II	Atezolizumab+Chemotherapy → cCRT → Atezolizumab	1-yr PFS: 78.1%	1-yr OS: 90.6%
InTRist^[28]^	II	Toripalimab+Chemotherapy → cCRT → Toripalimab	NR	-
PACIFIC BRAZIL^[29]^	II	Durvalumab+Chemotherapy → cCRT+Durvalumab → Durvalumab	1-yr PFS: 66.4%	-

NSCLC: non-small cell lung cancer; cCRT: concurrent chemoradiotherapy; sCRT: sequential chemoradiotherapy; mOS: median overall survival; mPFS: median progression-free survival; mrwPFS: median real-world progression-free survival; HR: hazard ratio; NR: not reached.

**表 2:** 不可切III期NSCLC放疗联合免疫正在进行的临床试验

Trial	Phase	Interventions	Primary endpoints	Status
PACIFIC-5^[30]^	III	cCRT/sCRT → Durvalumab	PFS	Active
PACIFIC-9^[31]^	III	cCRT → Durvalumab+Oleclumab/Monalizumab vs cCRT → Durvalumab	PFS	Recruiting
PACIFIC-8^[32]^	III	cCRT → Durvalumab±Domvanalimab	PFS	Recruiting
SKYSCRAPER-03^[33]^	III	cCRT → Atezolizumab+Tiragolumab vs cCRT → Durvalumab	PFS	Active
ECOG-ACRIN EA5181^[34]^	III	cCRT+Durvalumab → Durvalumab vs cCRT → Durvalumab	OS	Active
KEYLINK-012^[35]^	III	cCRT+Pembrolizumab → Pembrolizumab vs cCRT+Pembrolizumab → Pembrolizumab+Olaparib vs cCRT → Durvalumab	PFS, OS	Active

### 2.1 放化疗后的免疫巩固治疗

#### 2.1.1 同步放化疗（concurrent chemoradiotherapy, cCRT）+免疫巩固治疗

大型III期PACIFIC研究^[15,16]^评估了不可切除III期NSCLC患者在cCRT后进行度伐利尤单抗巩固治疗的有效性与安全性，其研究结论开创了III期NSCLC治疗新模式。结果显示，度伐利尤单抗组中位无进展生存期（median progression-free survival, mPFS）较对照组显著延长[16.9 *vs *5.6个月，风险比（hazard ratio, HR）=0.52，*P*<0.0001]，中位总生存期（median overall survival, mOS）同样显著延长（47.5 *vs* 29.1个月，HR=0.68，*P*=0.00251），且两组不良事件相似，免疫耐受性良好。5年随访数据^[16]^进一步证实联合度伐利尤单抗巩固治疗可带来持续生存获益（5年PFS率：33.1% *vs* 19.0%；5年OS率：42.9% *vs* 33.4%）。以PACIFIC研究的优秀数据为立足点，美国国立综合癌症网络（National Comprehensive Cancer Network, NCCN）和中国临床肿瘤学会（Chinese Society of Clinical Oncology, CSCO）指南均推荐度伐利尤单抗作为不可切除III期NSCLC患者接受cCRT后的巩固治疗（I级推荐）。另一项II期LUN 14-179研究^[17]^则探索帕博利珠单抗在cCRT后免疫巩固治疗中的临床价值，研究结果显示mPFS为18.7个月，mOS为35.8个月。

#### 2.1.2 序贯放化疗（sequential radiochemotherapy, sCRT）+免疫巩固治疗

虽然PACIFIC模式已成为不可切除III期NSCLC的标准推荐方案，但研究数据提示，仍有部分患者可能因为cCRT的毒性叠加而无法耐受^[36,37]^。因此sCRT作为无法耐受cCRT治疗患者的另一种选择，被推荐为可行的替代治疗方案。III期GEMSTONE-301研究^[18]^首次揭示国产舒格利单抗在cCRT/sCRT后III期NSCLC患者中的疗效，与度伐利尤单抗共同被纳入CSCO指南推荐。研究发现接受sCRT的127例患者中，舒格利单抗相较于安慰剂显著改善患者mPFS（8.08 *vs* 4.07个月），且各亚组间有一致获益趋势。单臂II期PACIFIC-6研究^[19]^临床获益与PACIFIC研究结果类似（3年OS率：56.5% *vs* 56.7%），但因其单独证实sCRT后度伐利尤单抗巩固治疗在III期NSCLC中的抗肿瘤活性，为sCRT作为cCRT不耐受患者的替代方案提供循证医学依据，同时期待大型III期PACIFIC-5研究^[30]^进一步验证。此外，在真实世界研究中，PACIFIC-R研究^[20]^探索了cCRT或sCRT后免疫巩固治疗效果，真实世界总体中位PFS（median real-world PFS, mrwPFS）为24.3个月，mOS达到59.0个月，5年mrwPFS率以及5年OS率分别为35.2%和49.2%，且无论cCRT或sCRT后接受度伐利尤单抗的患者均能长期获益（mrwPFS：25.8 *vs* 23.2个月；mOS：63.1 *vs* 47.1个月）。

#### 2.1.3 新型联合免疫巩固治疗

与此同时，不仅程序性细胞死亡受体1（programmed cell death protein 1, PD-1）抑制剂、PD-L1抑制剂备受关注，针对其他靶点的临床研究也在相继开展。COAST研究^[21,22]^旨在评估cCRT后度伐利尤单抗联合或不联合抗CD73单克隆抗体Oleclumab/抗NKG2A单克隆抗体Monalizumab在III期不可切除NSCLC中的疗效，研究结果示联合Oleclumab组或联合Monalizumab组的客观缓解率（objective response rate, ORR）分别为35.0%和40.3%，均高于度伐利尤单抗单药组（23.9%）。而且两联合组相较于单药组均有效延长mPFS（联合Oleclumab组：21.1 *vs* 7.3个月，HR=0.59；联合Monalizumab组：19.8 *vs* 7.3个月，HR=0.63），各组安全性相似。更新数据^[22]^表明联合Oleclumab、Monalizumab的2年OS率（76.8%, 72.1%）均较单药组（61.5%）显著延长，进一步巩固新药治疗优势。COAST研究的临床获益将进一步对III期PACIFIC-9研究^[31]^提供支持性数据，为III期NSCLC患者提供iRT模式下的治疗新方案。此外，为更深入探索放化疗后新型免疫联合方案，涉及T细胞免疫球蛋白ITIM结构域（T cell immune receptor with Ig and ITIM domain, TIGIT）这一新型ICIs的随机III期临床试验正在进行，即联合Domvanalimab和度伐利尤单抗的PACIFIC-8试验^[32]^，以及联合Tiragolumab和阿替利珠单抗的SKYSCRAPER-03试验^[33]^。

### 2.2 放化疗联合的免疫同步治疗

尽管PACIFIC模式已确立为不可切除III期NSCLC的标准治疗策略，然而部分患者仍因疾病进展或放化疗相关毒性等无法从免疫巩固治疗中获益。临床前研究^[38]^提示ICIs可通过增强肿瘤内CD8^+^ T细胞浸润改善局部肿瘤控制，这一机制为优化PACIFIC模式疗效及扩大获益人群提供新方向，促使研究者探索ICIs前移应用于III期NSCLC治疗的策略，以突破现有治疗瓶颈。ETOP NICOLAS研究^[23]^作为首次探索纳武利尤单抗联合cCRT后再免疫巩固治疗的单臂II期临床试验，成功验证该治疗模式的安全性并为后续不同药物研究奠定基础。与之类似的DETERRED研究^[24]^则证明该模式下阿替利珠单抗的临床价值（mOS：26.5个月* vs* 未达到，HR=0.71）。KEYNOTE-799试验^[25]^是另一项评估cCRT联合帕博利珠单抗后免疫巩固治疗在III期NSCLC中可行性的II期研究，队列A和B（仅含非鳞NSCLC）ORR均高于70%，mPFS分别为29.0和45.3个月，两队列mOS均显著延长（分别为35.6、56.7个月）。随后的2025年欧洲肺癌大会公布结果^[25]^显示，队列A和B两组2年PFS率和2年OS率分别为38.9%、46.3%以及40.2%、54.7%，提示该治疗方案能够改善III期NSCLC患者生存获益。上述II期临床试验虽然在样本量较小、缺乏对照组等方面存在局限性，但其数据仍为不可切除NSCLC患者的治疗提供新选择方案。由于II期研究初步获益，当前多项III期临床试验聚焦免疫放化疗联合免疫巩固方案的深入探索，包括ECOG-ACRIN EA5181研究^[34]^（cCRT联合度伐利尤单抗+度伐利尤单抗巩固）以及KEYLINK-012研究^[35]^（cCRT联合帕博利珠单抗+帕博利珠单抗巩固±Olaparib）。

### 2.3 放化疗前的免疫诱导治疗

近年来，放化疗前采用ICIs进行诱导的新模式能否为III期NSCLC患者带来临床获益，逐渐成为临床研究热点。AFT-16试验^[26]^是III期NSCLC中首个探索cCRT前ICIs（阿替利珠单抗）诱导的单臂多中心II期试验，数据显示该治疗方案mPFS可达30.0个月且2年PFS率提升至54.2%，尽管mOS未达到，但2年OS率为73.7%，显示出良好的抗肿瘤活性。2024年世界肺癌大会报道的APOLO研究^[27]^提供相似数据（1年PFS率为78.1%，OS率为90.6%）。InTRist研究^[28]^则将ICIs方案拓宽，探索诱导特瑞普利单抗联合化疗后cCRT免疫巩固治疗预后。与单纯化疗诱导组相比，免疫+化疗诱导组ORR（77.8% *vs *40.0%）和1年PFS率（89.4% *vs *57.8%）均显著改善，且毒性可控。随着以上临床研究证据积累，诱导免疫对不可切除III期NSCLC患者潜在关键价值日益突显，推动早期单臂II期研究逐步向多中心、大样本III期确证性研究转化。但需要强调的是单臂II期PACIFIC BRAZIL试验^[29]^结果与之相反，该研究评估强化诱导化疗与度伐利尤单抗治疗后，cCRT联合度伐利尤单抗巩固治疗III期NSCLC的疗效与毒性。结果显示1年PFS率有所改善，≥3级治疗相关不良事件总体发生率为82%，提示放化疗期间同时加入ICIs可能不必要，甚至会加重患者负担。以上研究均支持III期不可切除NSCLC患者在放化疗前用ICIs进行诱导，然而为进一步完善循证医学证据，亟需明确不良事件管理策略和治疗方案选择，同时期待大规模III期临床试验为该治疗方案提供可靠证据支持。

## 3 不可切除III期NSCLC的iRT的治疗思考

### 3.1 放疗剂量、分割方式的选择

随着肺癌精准治疗的快速发展，放疗剂量与分割方式作为影响疗效和安全性的关键因素，其最优方案尚未形成明确共识，仍需深入探索优化策略。RTOG 0617研究^[39]^结果表明，74 Gy高剂量组与60 Gy常规剂量组比较并未取得生存获益（mOS：20.3 *vs* 28.7个月，5年OS率：23% *vs* 32.1%），并增加放射性肺炎或心脏毒性等不良事件发生率。另一项随机III期临床研究^[40]^则显示，基于螺旋断层扫描疗法的中度低放疗可显著改善III期NSCLC者OS（3年OS率：58.4% *vs* 38.4%，mOS：41 *vs* 30个月，*P*=0.02），死亡风险降低49%。这些数据提示单纯盲目提高放疗剂量不值得推荐，合理优化放疗剂量以平衡疗效与毒性是改善III期NSCLC患者预后的关键。此外，多项研究围绕放疗分割模式或放疗方式展开深入探索，RG-LU004研究^[41]^显示加速分割放疗（60 Gy/15次）联合度伐利尤单抗方案的安全性同常规分割（60 Gy/30次）相当。另一项前瞻性II期GASTO-1049研究^[42]^证实大分割放疗后大分割加强联合每周同步化疗治疗模式的抗肿瘤活性，1和2年OS率分别为94.7%和72.4%。一项II期临床研究^[43]^和非随机对照试验^[44]^则共同聚焦立体定向放疗（stereotactic body radiotherapy, SBRT），III期NSCLC在含SBRT的不同放疗模式联合方案中呈现出良好耐受性且疗效得到强有力证实，为不可切除III期NSCLC治疗提供全新策略支持。同时多项回顾性分析^[45-47]^证明更先进的质子以及碳离子治疗技术能精准定向肿瘤靶病灶，减少射线对人体正常组织的损伤，进一步拓宽放疗受益人群。回顾以上临床研究数据，改变分割方式及放疗形式不仅显著缩短治疗时间还能减轻患者负担，为后续免疫巩固治疗创造潜在机会，但长期生存获益仍需验证。

### 3.2 ICIs的介入时间

不可切除III期NSCLC患者放化疗后ICIs的介入时机对其预后具有重要影响，是当前临床研究备受关注的关键问题。PACIFIC研究^[48,49]^事后探索性分析度伐利尤单抗介入时间与疗效获益关系，结果显示，放疗结束后2周内接受度伐利尤单抗治疗者，PFS和OS均显著优于放疗结束后超过2周后接受该方案患者（PFS: HR=0.39, OS: HR=0.42）。PACIFIC-R研究^[50]^在真实世界中进一步验证尽早介入度伐利尤单抗治疗的rwPFS获益优势（mrwPFS：25.7 *vs* 20.8个月）。同样DATE研究方案^[51]^规定III期NSCLC患者放化疗后立即予以度伐利尤单抗巩固治疗，1年PFS率可达75.0%且mPFS为14.2个月，不良事件耐受性良好，这些都证实放化疗后立即ICIs介入的可行性。II期LUN 14-179研究^[17]^也表现出与PACIFIC研究一致的疗效，但其亚组分析发现，cCRT 6-8周后接受ICIs者相较于4-6周后接受免疫巩固患者PFS和OS均具有临床获益趋势。多项*meta*分析研究^[52,53]^结果表明，以放疗后42 d为界值，早介入免疫巩固治疗与晚介入免疫巩固治疗患者1年PFS率无显著差异，不良事件发生风险也未见明显增加。以上分析表明不可切除III期NSCLC放化疗后ICIs巩固治疗的介入时间（≤14或≤42 d）均能带来临床获益且不良事件耐受良好。因此在临床实际工作中，对存在并发症风险患者适当延迟接受ICIs时间并不影响疗效。

### 3.3 联合治疗不良事件管理

iRT治疗策略在改善III期NSCLC患者受益的同时，带来的不良事件也显著增加，如何保留联合治疗的积极效应又避免不良事件的发生让患者获益最大化，亟需系统的规范化管理。小样本量II期DETERRED试验^[54]^数据显示，与cCRT组相比，sCRT未增加不良事件发生率（≥3级发生率：80% *vs *80%）。虽然大型III期GEMSTONE-301^[18]^和PACIFIC-R研究^[20]^均纳入cCRT及sCRT患者，但在安全性方面，未根据治疗时序进行毒性比较，仅分析总体耐受性。因此，对于不同治疗模式下的安全性比较，仍需更多数据支持。常见不良事件包括免疫相关性肺炎、放射性肺炎、皮肤反应和胃肠道毒性等，为正确评判其持续时间及严重程度，开始治疗前应进行体格检查、实验室检查和影像学检查，尤其是吸烟者、老年患者、合并自身免疫性疾病或肺基础疾病等高风险人群。需要强调的是，不仅基线需要常规评估，整个治疗期间都应密切监测患者情况以尽早发现潜在不良事件风险并采取相应治疗对策。发现不良事件时，需要严格评估标准以达到规范治疗目的，常按照不良事件通用术语标准（Common Terminology Criteria for Adverse Events, CTCAE）对毒性反应进行分级^[55]^。除对已发生事件进行对应处理外，还应在发生前采取适当预防性干预措施，如对放疗计划合理优化，预防性使用低剂量糖皮质激素，进行生物标志物动态监测，设计相关模型预测发生概率等个体化策略。此外还推荐包括但不限于涵盖肿瘤内科、呼吸科、放疗科、影像科、药学部在内的多学科诊疗（multidisciplinary therapy, MDT）模式，共同参与联合治疗的毒性评估与管理。但不管最终采用何种管理方式，能达到最大治疗效益的模式都值得肯定。

### 3.4 生物标志物的探索

尽管iRT治疗模式已成为标准治疗方案，但因治疗反应存在异质性，部分患者未能获益。因此，亟需寻找能预测疗效、规避风险并筛选潜在获益人群的生物标志物，以制定个体化治疗策略。

PD-L1表达水平作为近年来研究最为深入且最受认可的生物标志物之一，其预测ICIs治疗预后的能力备受关注^[56]^。大型回顾性RELEVANCE研究^[57]^结果显示，PD-L1≥50%、1%-49%和<1%患者的3年OS率分别为69%、44%和39%，高表达组（≥50%）死亡风险显著低于阴性组（<1%）。PACIFIC-R研究^[50]^亚组分析同样证实PD-L1≥1%患者较PD-L1<1%具有更好临床获益（mPFS：22.4 *vs *15.6个月）。值得注意的是，PACIFIC探索性分析^[58]^提示PD-L1<1%患者未观察到OS获益，而基于GEMSTONE-301^[18]^结果，CSCO指南建议无论PD-L1状态均可接受免疫巩固治疗。这些矛盾数据表明，PD-L1表达与生存获益虽存在正相关趋势，但PD-L1表达阴性患者能否获益仍存争议，可能与放疗模式、肿瘤异质性及免疫微环境等因素有关。

基于循环肿瘤DNA（circulating tumor DNA, ctDNA）的微小残留病灶（minimal residual disease, MRD）检测是另一重要研究方向。DART研究^[59]^证实，cCRT后4个月内检测到ctDNA阳性患者的2年死亡风险显著增加[比值比（odds ratio, OR）=16.48, *P*=0.017]。BTCRC LUN 16-081试验^[60]^发现，与MRD阴性者相比，完成cCRT后MRD阳性患者生存率明显降低（2年PFS率：65% *vs *29%）。前瞻性研究^[61]^表明残留ctDNA患者的mPFS缩短约18个月（HR=3.303）。值得注意的是，该人群接受免疫巩固治疗仍可显著获益（mPFS：13.8 *vs *6.2个月，*P*=0.028；mOS：未达到 *vs *14.8个月，*P*=0.005）。这些证据共同提示，ctDNA阳性是复发高危标志，同时可精准筛选ICIs治疗获益人群。

影像组学作为无创评估工具，在风险分层中展现独特价值。一项回顾性研究^[62]^显示，高影像组学评分组的mOS显著优于低评分组（24.3 *vs *11.5个月，HR=0.58，*P*=0.0081）。另一项研究^[63]^则证实，无论PD-L1水平如何，高危影像组学特征均可独立预测PFS（*P*<0.0001）。一系列研究^[64,65]^表明，影像组学还可以预测放射性肺炎风险，其受试者工作特征曲线下面积（area under the curve, AUC）为0.76-0.85，并可鉴别放射性肺炎与免疫性肺炎（AUC: 0.81-0.86）。但当前各影像组学模型存在标准不统一、验证不足等局限，未来还需通过前瞻性临床试验结合个体化临床判断进行验证和优化。

除上述标志物外，肿瘤突变负荷^[66]^、错配修复缺陷^[67]^、微卫星不稳定性^[68]^、细胞游离RNA^[69]^、干扰素-γ相关基因^[70]^及肿瘤浸润淋巴细胞^[71]^等均为潜在生物标志物。未来研究需验证其临床适用性，并通过多维度标志物联合应用提升预测效能。

## 4 总结与展望

iRT在不可切除III期NSCLC领域已取得革命性突破，以PACIFIC研究为代表的循证证据确立了放化疗后免疫巩固治疗的标准地位，但临床实践仍面临关键挑战。目前，放疗最佳剂量与分割模式尚未形成明确共识，ICIs介入的最佳时机也有待进一步探索和验证。无论是何种iRT模式，都必须重视治疗相关不良事件的个体化精细防控和有效管理。未来，我们也应通过MDT与一系列前瞻性临床试验的开展，继续深入探索iRT的优化策略，同时构建多组学整合预测模型以精准预测iRT的疗效与毒性，最终以期实现个体化的精准治疗，改善患者的长期生存。
